# A Phylogenomic View of Ecological Specialization in the Lachnospiraceae, a Family of Digestive Tract-Associated Bacteria

**DOI:** 10.1093/gbe/evu050

**Published:** 2014-03-12

**Authors:** Conor J. Meehan, Robert G. Beiko

**Affiliations:** ^1^Department of Biochemistry and Molecular Biology, Dalhousie University, Halifax, Nova Scotia, Canada; ^2^Faculty of Computer Science, Dalhousie University, Halifax, Nova Scotia, Canada

**Keywords:** lateral gene transfer, microbial genomes, metagenomics, phylogenomics, butyric acid, sporulation

## Abstract

Several bacterial families are known to be highly abundant within the human microbiome, but their ecological roles and evolutionary histories have yet to be investigated in depth. One such family, Lachnospiraceae (phylum Firmicutes, class Clostridia) is abundant in the digestive tracts of many mammals and relatively rare elsewhere. Members of this family have been linked to obesity and protection from colon cancer in humans, mainly due to the association of many species within the group with the production of butyric acid, a substance that is important for both microbial and host epithelial cell growth. We examined the genomes of 30 Lachnospiraceae isolates to better understand the origin of butyric acid capabilities and other ecological adaptations within this group. Butyric acid production-related genes were detected in fewer than half of the examined genomes with the distribution of this function likely arising in part from lateral gene transfer (LGT). An investigation of environment-specific functional signatures indicated that human gut-associated Lachnospiraceae possess genes for endospore formation, whereas other members of this family lack key sporulation-associated genes, an observation supported by analysis of metagenomes from the human gut, oral cavity, and bovine rumen. Our analysis demonstrates that adaptation to an ecological niche and acquisition of defining functional roles within a microbiome can arise through a combination of both habitat-specific gene loss and LGT.

## Introduction

Mammal-associated microbiomes have been shown to influence host health and behavior (Cryan and O’Mahony 2011; Kinross et al. 2011; Muegge et al. 2011) and appear to be hotbeds for lateral gene transfer (LGT) (Smillie et al. 2011; Meehan and Beiko 2012). Lachnospiraceae is a family of clostridia that includes major constituents of mammalian gastrointestinal (GI) tract microbiomes, especially in ruminants (Kittelmann et al. 2013) and humans (Gosalbes et al. 2011). The family is currently described in the National Center for Biotechnology Information (NCBI) taxonomy as comprising 24 named genera and several unclassified strains (Sayers et al. 2010) that share 16S ribosomal RNA gene (henceforth referred to as 16S) similarity (Bryant 1986; Dworkin and Falkow 2006). All known family members are strictly anaerobic (Dworkin and Falkow 2006), reside mainly within the digestive tracts of mammals (Bryant 1986; Downes et al. 2002; Carlier et al. 2004; Moon et al. 2008), and are thought to be primarily nonspore forming (Dworkin and Falkow 2006). Several members play key roles within the human GI microbiome, demonstrated by their inclusion in an artificial bacterial community that has been used to repopulate a gut microbiome and remedy *Clostridium difficile* infections (Petrof et al. 2013). Early blooms of Lachnospiraceae may be linked with obesity (Cho et al. 2012), most likely due to their short-chain fatty acid (SCFA) production (Duncan et al. 2002). However, despite their apparent importance, little is known about the group as a whole outside of its use as an indicator of fecal contamination in water and sewage (Newton et al. 2011; McLellan et al. 2013) and the abundance of butyric acid-producing species within the group (Bryant 1986; Duncan et al. 2002; Louis et al. 2004, 2010; Charrier et al. 2006).

Butyric acid (also known as butanoic acid, butanoate, and butyrate) is an SCFA whose production prevents the growth of some microbes within the digestive tract (Zeng et al. 1994; Sun et al. 1998) and provides a source of energy for other microbes (Liu et al. 1999) and host epithelial cells (Roediger 1980; McIntyre et al. 1993; Hague et al. 1996; Pryde et al. 2002). Butyrate also regulates expression of the AP-1 signaling pathway in key components of human physiology (Nepelska et al. 2012). These functions link butyric acid to protection against colon cancer (Hague et al. 1996; Mandal et al. 2001) and a potential influence on obesity levels (Duncan et al. 2008; Turnbaugh et al. 2008). Two pathways are responsible for fermentation of this SCFA: through butyrate kinase or through butyryl-CoA:acetate CoA-transferase (BCoAT) (Walter et al. 1993; Duncan et al. 2002). This production appears to be restricted mainly to organisms within the class Clostridia (Louis et al. 2010) and has been demonstrated in many strains of Lachnospiraceae (Attwood et al. 1996; Duncan et al. 2002; Charrier et al. 2006; Kelly et al. 2010; Louis et al. 2010).

Although the production of butyrate links many Lachnospiraceae to key roles within digestive tract microbiomes, it is not known why only some members produce this SCFA and what the ecological role of the remaining family members might be. Here, we investigate the relationship between phylogeny, ecology, and biochemistry in this group by examining a set of 30 sequenced genomes, combined with marker gene surveys from a wide range of habitats and metagenomic samples collected from the habitats with high numbers of Lachnospiraceae. Endospore formation distinguished Lachnospiraceae from different habitats, with complete or near-complete sporulation pathways in human gut-associated microorganisms, and many key pathways absent from other members of the group. Although endospore formation capability appeared to be a result of habitat-specific loss, the distribution of butyrate production capabilities showed strong evidence of LGT. The fluidity of butyrate production and other properties highlights a range of evolutionary processes that impact on adaptation and host interactions.

## Materials and Methods

### Assessing the Habitat of Lachnospiraceae Members

A determination of the environmental range of members of the Lachnospiraceae was undertaken using a phylogenetic assignment method. All 16S sequences from completed genomes and all Clostridiales-type strains in the Ribosomal Database Project (Cole et al. 2009) were aligned to the Greengenes reference alignment template using PyNAST (Caporaso et al. 2010) and masked to include only the phylogenetically informative sites, resulting in an alignment of 2,217 sequences and 1,287 sites. A reference tree was then created from these sequences using RaxML version 7.2.5 (Stamatakis 2006) with a GTR + Γ model. Presence within a habitat was assessed by aligning reads from 1,697 environmental samples of 16S sequences from MG-RAST (Meyer et al. 2008), sorted into 17 habitat types (supplementary table S1, Supplementary Material online), added to the reference alignment using PyNAST, and placed on the reference tree using pplacer version 1.1.alpha13 (Matsen et al. 2010). Taxonomic classification of reads was then undertaken using the classify function of guppy, a part of the pplacer package. Reads were classified as a given taxonomic rank if the posterior probability of that assignment was above 0.7. The percentage of classified reads assigned to Lachnospiraceae was calculated per sample and then aggregated between samples into broad habitat definitions.

### Butyric Acid Production

Sequenced genomes identified as Lachnospiraceae were retrieved from NCBI on April 18, 2012 (supplementary table S2, Supplementary Material online). This resulted in 30 genomes (2 completed and 28 permanent draft) from four primary habitats: the human digestive tract, cow rumen, human oral cavity, and sediment containing paper-mill and domestic waste. The potential for butyric acid production was then assessed within each Lachnospiraceae sequenced genome. Sequences annotated as butyrate kinase were retrieved from the KEGG database, version 58.1 (Kanehisa et al. 2004), as this encodes one of the final steps of the two butyric acid pathways. The other path to butyric acid production is through utilization of BCoAT (Louis et al. 2010). The sequences derived from Louis et al. (2010) constituted the reference database for our search. These two data sets were used to mine the protein sets of each sequenced Lachnospiraceae genome using USEARCH 4.0.38 (Edgar 2010) with an *e*-value cut off of 10^−^^30^ and a minimum identity cut off of 70%. The origin of the butyrate-related genes was assessed using a phylogenetic approach. Protein sequences encoded by 3,500 bacterial and archaeal genomes were retrieved from NCBI, and USEARCH was used in same manner as above to search for the two butyric acid-related genes, with the Lachnospiraceae sequences identified above as queries. Sequences were aligned using MUSCLE version 3.8.31 (Edgar 2004a, 2004b) and trimmed using BMGE version 1.1 (Criscuolo and Gribaldo 2010) with a BLOSUM30 matrix and a 0.7 entropy cut off. A phylogenetic tree was created using FastTree version 2.1.4 (Price et al. 2010) with a GTR model and a gamma parameter to model rate variation across sites.

To test whether LGT occurred within the history of these genes, a comparison of the resulting topologies to the 16S tree (as a proxy for implied vertical inheritance) was undertaken. The longest 16S sequence from each genome found to have a predicted butyrate kinase was extracted, and an alignment and tree were built as above. The per-site likelihoods of the 16S topology and the topology based on the butyrate kinase alignment were calculated using FastTree with the butyrate kinase alignment as the data set, and an approximately unbiased (AU) test was performed using CONSEL (Shimodaira and Hasegawa 2001). This procedure was repeated using the BCoAT-containing species.

### Clustering of Genomes Based on Homologous Gene Groups

A comparative genomics approach was undertaken to understand the shared functional repertoires of members of the Lachnospiraceae. To construct a set of shared homologous protein-coding genes, BLASTClust (Altschul et al. 1990) was employed with a minimum match criterion of 40% identity and 70% length on all genes. Functional assignment to each cluster was performed using the Clusters of Orthologous Groups (COG) database (Tatusov et al. 2000). BlastP (Altschul et al. 1990) with a 10^−^^3^ *e*-value cut off was employed for each gene cluster using representative protein sequences for each of the 18 COG functional categories as a database. Lachnospiraceae genomes were then clustered based on pair-wise counts of shared homologous gene clusters to look for associations between shared genome content and habitat. These pair-wise counts were calculated using a normalized Hamming distance, such that the distance between genomes *x* and *y* is (*A* + *B* − 2*S*)/(*A* + *B*) where *A* and *B* are the total gene counts of *x* and *y,* respectively, and *S* is the number of shared genes between *x* and *y* (Lin and Gerstein 2000). If a cluster contained more than one gene in a given genome (e.g., in-paralogs), *S* equals the smaller gene count per genome. Counts were then clustered and displayed using the R package gplots (Warnes et al. 2012). Groups of interest were further analyzed using Interproscan version 4.8 (Zdobnov and Apweiler 2001) to determine what functions may define such groups.

### Distribution of Sporulation Capabilities in Sequenced Genomes and Metagenomes

Each Lachnospiraceae genome was compared with the sporulation-associated proteins as found within *Bacillus subtilis* strain 168 (Kunst et al. 1997). The *B. subtilis* proteins labeled as within the main sporulation-associated families *cot*, *spol*, *sps*, and *ssp* were used as a database for a BlastP search with a 10^−30^ *e*-value cut off and all Lachnospiraceae proteins as queries. The putative history of each sporulation protein was assessed with the same phylogenetic method as was used for the butyric acid-related proteins.

Metagenomes for the human digestive tract (Yatsunenko et al. 2012; MG-RAST project 401; 107 samples), human oral cavity (Human Microbiome Project; MG-RAST project 385; 12 samples), and cow rumen (Brulc et al. 2009; MG-RAST project 24; four samples; Hess et al. 2011; SRA023560; one sample) were used to assess the distribution of Lachnospiraceae-derived sporulation proteins in culture-independent data sets. The Lachnospiraceae-associated sporulation genes were used as a database with a metagenome sample as a query input to USEARCH with a 10^−^^10^ *e*-value cut off. From this set of results, we removed all reads whose best match was to a non-Lachnospiraceae genome in the set of 3,500 NCBI genomes. Final counts of reads designated as sporulation-associated were compared between habitats using STAMP version 2 (Parks and Beiko 2010) with a two-sided Welch’s *t*-test and Bonferroni multiple test correction.

### Phylogenomic Analysis of the Lachnospiraceae

Assessment of intra-family relationships was undertaken using three different methods: phylogenetic tree inference using 16S, tree inference using a concatenated alignment of 91 shared protein-coding genes, and a consensus network of relationships (Holland et al. 2004) based on the same set of shared genes.

All 16S rRNA gene sequences over 1,000 nucleotides long from each Lachnospiraceae genome along with those of two species from the family Ruminococcaceae as an outgroup (*Ruminococcus albus* 7 and *Ethanoligenens harbinense* YUAN-3) were aligned using PyNAST and trimmed to include only the phylogenetically informative sites used by Greengenes (DeSantis et al. 2006). A reference tree was created using RaxML version 7.2.5 with the evolutionary model GTR + Γ + I as selected using the Bayesian information criterion in PartitionFinder (Lanfear et al. 2012). A set of family-wide shared genes was created from the homologous gene clusters output from BlastClust. Those gene sets that were present as a single copy in each genome were selected, and orthology was confirmed using an all versus all BlastP between Lachnospiraceae proteomes with an *e* value of 10^−^^10^. USEARCH was used with an *e*-value cut off of 10^−^^30^ to find genes in the completed genomes of members of the Ruminococcaceae that would serve as an outgroup to these family-wide genes. Alignments were constructed using MUSCLE and trimmed using BMGE as above. Resulting alignments were then concatenated and a tree inferred using FastTree with a gamma parameter.

SEQBOOT (Felsenstein 1989) was used to generate 100 randomizations each of the 16S and concatenated alignments, which were then subjected to phylogenetic analysis as above to establish bootstrap support. Concordance between the 16S tree and concatenated alignment tree was tested using the subtree prune-and-regraft (SPR) distance with rSPR version 1.2.0 (Whidden et al. 2010) and the AU test in CONSEL version 0.20 (Shimodaira and Hasegawa 2001). Individual gene alignments were also tested for concordance with the concatenated sequence tree using the AU test in CONSEL and with each other using rSPR. The set of shared Lachnospiraceae protein-coding genes was used to create a consensus network using SplitsTree4 (Huson and Bryant 2006) with a 0.7 similarity cut off and edges weighted by counts.

## Results

### Lachnospiraceae Are Common Only in Host-Associated and Sewage Effluent Samples

**Fig. 1.—:**
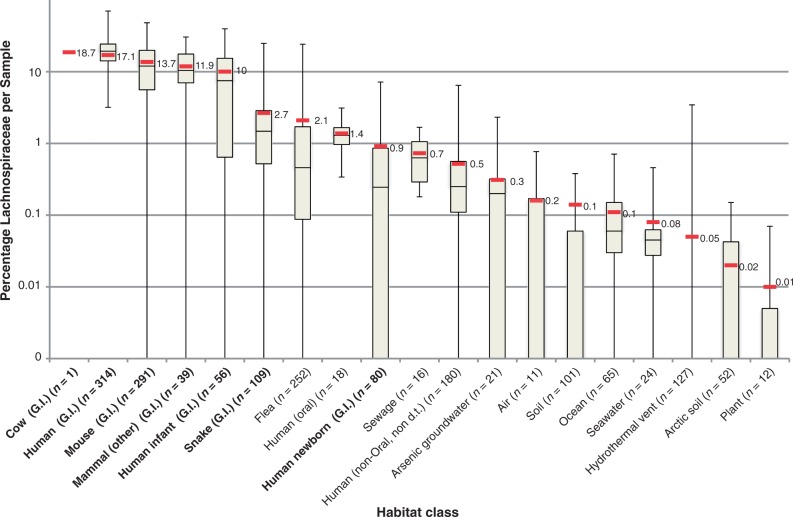
Environmental distribution of the Lachnospiraceae. A total of 25 16S rRNA gene surveys containing a total of 1,697 samples covering 17 different habitat classes were taxonomically profiled to identify the overall percentage of Lachnospiraceae. Boxplots outline the 25th, 50th, and 75th percentiles of the data. The minimum, maximum, and average (red box) percent abundance per sample of this family are also indicated. The number of samples per environment is listed beside habitat type and in supplementary table S1, Supplementary Material online. Each GI tract-associated habitat is highlighted in bold.

We examined a total of 1,697 published marker gene surveys from different environments to determine the primary habitats of the Lachnospiraceae. Sequences associated with the group were more abundant in the GI tracts of mammals compared with other environments, including other mammal-associated body sites (fig. 1). Although mammalian GI samples tended to have a relative abundance of Lachnospiraceae in excess of 10%, in others the relative abundance was often less than 1%. Variation was found between different human life stages with abundance of Lachnospiraceae highest in the adult GI tract, moderate in infants, and approximately 1% in newborns. Smaller proportions were found in other animals such as fleas and snakes, whose numbers were higher than those of newborn humans and all nonanimal-associated habitats. Only the cow rumen, human digestive tract, human oral cavity, and sewage effluent microbiomes were predicted to have Lachnospiraceae in every sample. As Lachnospiraceae genomes from similar environments were available, extensive functional and phylogenomic analysis of the group was undertaken using 30 representative genomes (supplementary table S2, Supplementary Material online).

### Butyric Acid Production Is Not a Defining Trait of the Lachnospiraceae

Lachnospiraceae members have been implicated in butyric acid production in the human GI tract (Duncan et al. 2008; Louis et al. 2010; Van-den-Abbeele et al. 2012). It is known that not all members of this family can produce butyrate, raising the question of whether this function was acquired laterally or was ancestrally present and then lost in several lineages. Here, the capability to produce butyric acid along with its evolutionary history was investigated to determine its distribution within the group and the origin of associated genes. Two enzymes allow for the production of butyric acid: butyrate kinase (from Butanoyl-P) (Walter et al. 1993) and BCoAT (from Butanoyl-CoA) (Duncan et al. 2002). Only 12 of the 30 sequenced organisms contained genes annotated from at least one of these two pathways (table 1). Pathways appeared to be genus specific as all *Shuttleworthia, Butyrivibrio,* and *Coprococcus* genomes encode butyrate kinase and both *Roseburia* strains, both *Anaerostipes* strains, and *Lachnospiraceae* bacterium 5_1_63FAA encode BCoAT. Analysis with TBlastN did not reveal any hits within the other Lachnospiraceae genomes, indicating no related pseudogenes are within these species.

**Table 1:** The Distribution of Butyric Acid Production Genes

Name	Butyrate Kinase	BCoAT
*Anaerostipes caccae* DSM 14662		+
*Anaerostipes* sp. 3_2_56FAA		+
*Butyrivibrio crossotus* DSM 2876	+	
*Bu. proteoclasticus* B316	+	
*Catonella morbi* ATCC 51271		
*Cellulosilyticum lentocellum* DSM 5427		
*Coprococcus comes* ATCC 27758	+	
*Co. eutactus* ATCC 27759	+	
*Dorea formicigenerans* ATCC 27755		
*D. longicatena* DSM 13814		
*Lachnospiraceae* bacterium 1_1_57FAA		
*Lachnospiraceae* bacterium 1_4_56FAA	+	
*Lachnospiraceae* bacterium 2_1_46FAA		
*Lachnospiraceae* bacterium 2_1_58FAA		
*Lachnospiraceae* bacterium 3_1_46FAA		
*Lachnospiraceae* bacterium 3_1_57FAA_CT1	+	
*Lachnospiraceae* bacterium 4_1_37FAA		
*Lachnospiraceae* bacterium 5_1_57FAA		
*Lachnospiraceae* bacterium 5_1_63FAA		+
*Lachnospiraceae* bacterium 6_1_63FAA		
*Lachnospiraceae* bacterium 8_1_57FAA		
*Lachnospiraceae* bacterium 9_1_43BFAA		
*Lachnospiraceae* oral taxon 107 str. F0167		
*Marvinbryantia formatexigens* DSM 14469		
*Oribacterium sinus* F0268		
*Oribacterium* sp. oral taxon 078 str. F0262		
*Oribacterium* sp. oral taxon 108 str. F0425		
*Roseburia intestinalis* L1-82		+
*R. inulinivorans* DSM 16841		+
*Shuttleworthia satelles* DSM 14600	+	

Note.—The final stage of butyric acid production can be undertaken by two gene groups: butyrate kinase or BCoAT. The presence of each gene within a Lachnospiraceae genome is marked with a +.

Phylogenetic examination of the two genes revealed evidence of potential LGT. Genes similar to those of the Lachnospiraceae were found within the genomes of several taxa, primarily of the same order as the Lachnospiraceae, the Clostridiales. The topology of each gene tree was tested against a 16S tree derived from the same genomes (supplementary fig. S1, Supplementary Material online). Use of the AU test (based on the butyrate kinase and BCoAT alignments) showed that the gene trees for butyrate kinase and BCoAT in these species were significantly different from the companion 16S tree (*P* < 0.001). This implies that rearrangements away from a proxy for vertical inheritance occurred within the gene trees, indicative of LGT of both butyrate kinase and BCoAT. Additionally, the 16S tree placed many species that are not currently classed in the NCBI taxonomy as Lachnospiraceae (e.g., *Eubacterium rectale*) proximal to recognized members of this family, suggesting the need for taxonomic revision of the group.

### Shared Gene Clusters Reveal Functional Signatures of Habitat Specialization

A thorough investigation of the family was undertaken to look for defining features of the Lachnospiraceae using sets of homologous gene clusters shared between members of this bacterial family. A total of 167 gene clusters were shared by all sequenced Lachnospiraceae with predicted functions spanning information processing (46%), metabolism (15%, primarily glycolysis and fructose metabolism; COG category G), and cellular processes/signaling (9%), including two multidrug resistance mechanisms and several sigma factors. Thus, only 16S similarity and a handful of metabolic and cellular processes appear to be shared by all members of the Lachnospiraceae family.

**Fig. 2.—:**
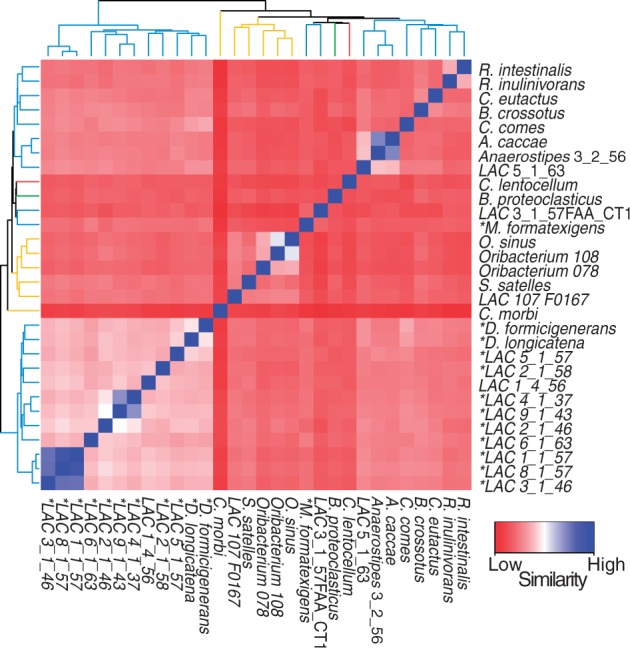
Grouping of genomes based on counts of shared gene clusters. Heatmap shows the number of gene clusters shared between genomes, inversely weighted by genome size. Genomes are clustered with intersecting cells between two genomes colored based on similarity ranging from low (red) to high (blue). The hierarchy of clustering is displayed along the side and top of the heat map with branches colored according to habitat (yellow, oral; red, sediment; green, rumen; blue, human GI tract). Names of gut-associated members predicted to be lacking butyric acid production are highlighted by an asterisk.

Ecological specialization was investigated using pair-wise gene cluster counts between sequenced genomes to observe whether habitat correlated with the presence of specific groups of genes (fig. 2). Some association between habitat and clustering was observed, including a basal split into a group consisting exclusively of 12 human gut-associated family members (referred to as the gut-restricted group) and another group containing genomes from all represented habitats, which contained a smaller cluster of eight gut-associated genomes (fig. 2). The average genome size was 3,539 genes (range: 1,950–6,887) for the mixed habitat group and 2,920 genes (range: 2,081–3,534) for the gut-restricted group. The average genome size within this data set, regardless of clustering, is 3,291, suggesting that group associations are not biased by genome size.

Gene clusters that defined certain groups were investigated further to observe functional patterns. A gene cluster was classed as group specific if it was present in at least 90% of the genomes in one group and absent from 90% of the complementary group. Comparison of the gut-restricted group and all other Lachnospiraceae revealed 41 shared gene clusters that were indicative of this group (i.e., present in at least 11 gut-restricted genomes and absent from at least 16 of the other genomes). Functionally, these genes encompassed mostly protein binding (primarily tetratricopeptide repeat motifs), signal transduction, and sporulation, with almost a third of the homologous gene clusters having no annotated function (supplementary table S3a, Supplementary Material online). Only one gene cluster, annotated as an inner membrane component of a transporter complex, was classed as a defining gene cluster for the multihabitat group when compared with the gut-restricted group.

The gut-restricted group was also found to have several gene clusters that distinguish them from the 8 genomes of the other gut-associated Lachnospiraceae (supplementary table S3b, Supplementary Material online). Several tetratricopeptide repeat protein-binding motifs were present in the gut-restricted group and absent from many of the other gut-associated genomes. Most other potentially defining functions encompassed transporters and signaling pathways with 30% of clusters having no known function. The reverse comparison (clusters absent from the majority of the gut-restricted group but present in the other gut-associated members) revealed several catalytic and transportation-related functions without any discernible pattern.

Almost all of the organisms in the gut-restricted group were also those predicted to be incapable of producing butyric acid (supplementary table S4, Supplementary Material online; fig. 2). This indicates a split in the human gut-associated Lachnospiraceae between those capable of producing butyric acid by either of the known pathways and those who, while lacking this capability, have genomes that are more closely related to each other (the gut-restricted group). Several gene clusters that correlated with the presence or absence of butyric acid production within the human GI-tract-associated Lachnospiraceae (supplementary table S3c, Supplementary Material online) also distinguished the gut-restricted group from the other gut-associated family members (supplementary tables S3b and table S4, Supplementary Material online). Thus, even though multiple pathways can result in butyric acid production, the presence or absence of this function appears to have an influence on the specialization of certain organisms within the human gut microbiome.

To observe whether similar patterns of distinguishing functions existed between all gut-associated family members (22 strains) and nongut associated members (eight strains), a similar analysis of gene group presence/absence was performed. Fifty-seven functions present in 20 or more gut-associated strains and absent from seven or eight nongut-associated strains were identified (supplementary table S3d, Supplementary Material online). Only one protein was of unknown function with the remaining spread across designations such as DNA binding, repair, and transcription. Several serine-type endopeptidases or associated proteins were present within this group and lacking from the others, suggesting potential involvement of protein modification in adaption to the human GI tract environment. As was observed with the gut-restricted group, sporulation-related proteins comprised a large fraction of these functions (28%), although different sporulation proteins distinguished these two groups.

### Key Sporulation Proteins Are Detected Only in Human Digestive Tract-Associated Family Members

**Fig. 3.—:**
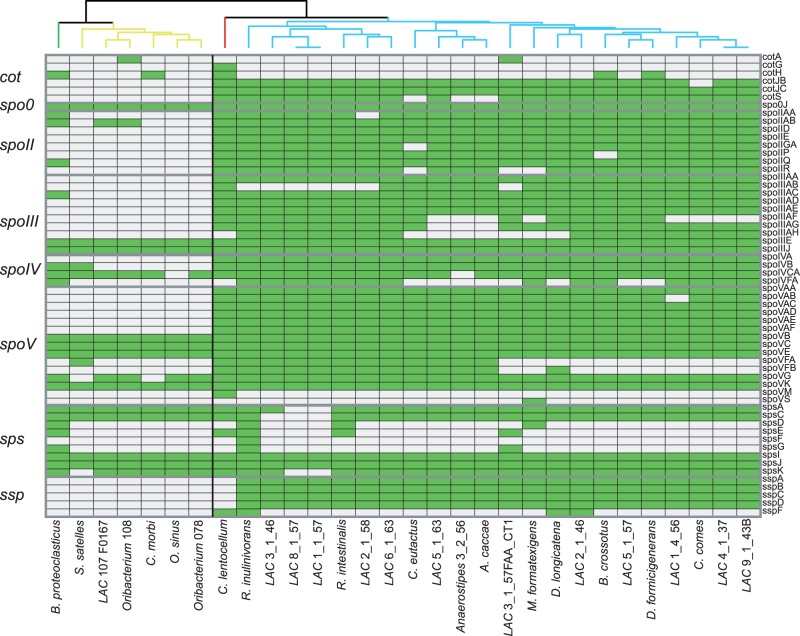
Distribution of sporulation-associated genes within Lachnospiraceae genomes. A range of sporulation genes was examined for each genome to assess the capabilities of producing endospores within each strain. Each gene is displayed as present (green) or absent (white) from each Lachnospiraceae genome. Organisms are clustered based on their distribution of sporulation genes. Hierarchical clustering of genomes is displayed at the top of the grid with branches colored according to habitat (yellow, oral; red, sediment; green, rumen; blue, human GI tract). Gray lines separate sporulation genes into the broad categories listed on the right-hand side.

We further examined the distribution of four types of sporulation genes: *cot* genes, which encode protein components of the coat; *spo* genes, which perform functions across all six stages of sporulation; *sps* genes, involved in spore coat polysaccharide synthesis; and *ssp* genes, which create small acid-soluble spore proteins. Homology searches against related sequences from *B. subtilis* (84 genes) revealed that 27 of these genes had no known homolog in any Lachnospiraceae sequenced genome. Of the remaining 57 genes, 29 were present in the majority of gut-associated Lachnospiraceae and completely absent from the rumen and oral-associated family members (fig. 3). These genes were not restricted to any one class or stage of sporulation protein. *Cellulosilyticum lentocellum* DSM 5427, isolated from sediment containing domestic waste, grouped with the gut-associated members suggesting that it too may be adapted to the human digestive tract.

All sporulation-controlling sigma factors (σ^A^, σ^E-H^, and σ^K^) were detected in all Lachnospiraceae genomes, which suggests this function was present in the ancestor of the group. Phylogenetic analysis also suggested vertical transmission of this function, although uncertain taxonomic assignments make such conclusions difficult to confirm. These analyses suggest that gene loss rather than LGT is responsible for the observed habitat-associated pattern of sporulation genes. To confirm a differential presence of sporulation capability in the three habitats (human gut, human oral cavity, and cow rumen), metagenomic samples from each microbiome were mined to find sequences related to each Lachnospiraceae-associated sporulation protein. Lachnospiraceae-derived sporulation-associated reads were found to be more abundant within the human GI tract compared with the cow rumen or human oral cavity (*P* < 0.001) (supplementary fig. S2, Supplementary Material online). The difference in abundance between the rumen and oral cavity was less well supported (*P* = 0.022; difference in relative means = 0.013). Thus, it is likely that sporulation capabilities within this family are restricted to those found in the human GI tract.

### Candidate Phylogenies Do Not Reflect Habitat Diversification

**Fig. 4.—:**
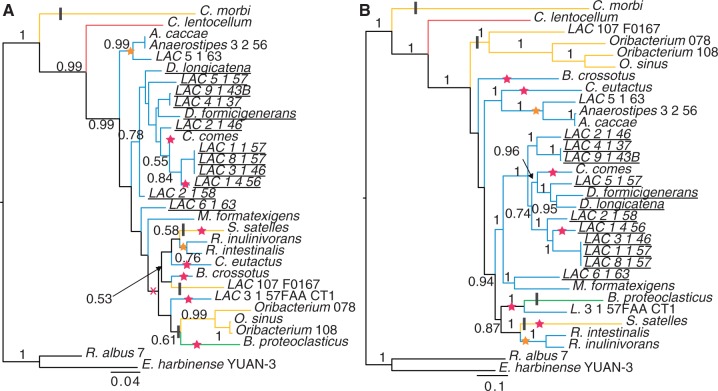
Relationships of 30 Lachnospiraceae genomes based on marker gene and concatenated alignments. Phylogenetic trees based on the 16S ribosomal RNA gene (*A*) and the family-wide shared orthologs (*B*). Trees are rooted using two Ruminococcaceae as outgroup. Branches are colored based on listed habitat (yellow, oral; red, sediment; green, rumen; blue, human GI tract). Bootstrap support values greater than 0.5 are displayed. Locations of putative gain and loss of functions are also shown on the trees. Stars mark the gain of butyric acid production capabilities (pink, butyrate kinase; orange, BCoAT). An alternative gain of butyrate kinase is marked with a pink X on the 16S tree (part A). Putative loss of sporulation capabilities is marked with a black bar. Strains classified as gut restricted based on shared gene clusters are underlined.

Functional analysis of the Lachnospiraceae-associated genomes revealed both vertical and lateral acquisition of genes that were indicative of subgroups within the family. Although species tree reconstruction can be undertaken in several ways, we chose two popular methods for comparison: 16S phylogeny and a shared ortholog concatenated alignment phylogeny. The copy number of the 16S rRNA gene ranged from 1 (22 genomes) to 11 (one genome) with an average of 2.1 copies per genome. Despite this diversity in copy number, all 16S sequences formed clades with respect to their genome. Therefore, only a single representative (the copy with the longest sequence) was retained within the final phylogenies (fig. 4*a*). The 16S tree yielded little support for the majority of clades (38% of clades with >75% bootstrap support) (fig. 4*a*), likely due to short internal branches in the tree (Wiens et al. 2008). This poor support contrasted with strong support across the tree derived from the concatenated alignment from 91 ubiquitous, single-copy orthologous genes (88% of clades with >75% bootstrap support) (fig. 4*b*). However, this tree was not in strong agreement with those of the 91 constituent genes according to the AU test (82% rejected with *P* < 0.001). Even within this restricted “core” set shared by all family members (supplementary table S5, Supplementary Material online), significant phylogenetic discordance is observed. Comparisons of individual core set gene trees revealed low agreement, with only 47 of the 91 gene trees being within an SPR distance of 2 from at least one other shared ortholog tree (supplementary fig. S5, Supplementary Material online). No large core set of genes was found to be in high agreement with each other, suggesting that even core genes are subject to phylogenetic discordance. Comparison of phylogenetic relationships derived from 16S sequences and concatenated shared orthologs revealed substantial topological differences, as demonstrated by an SPR distance of 12 between trees with only 30 leaves (supplementary fig. S3, Supplementary Material online). Additionally, each tree was rejected under the AU test (*P* < 0.001) when compared with the alignment of the other (i.e., 16S topology derived from the concatenated alignment and vice versa), demonstrating that neither the 16S tree nor the concatenated alignment tree is a convincing proxy for the evolutionary history of the full genomes. The consensus network based on the 91 shared orthologs demonstrated that no clear signal could differentiate the majority of individual strains into a hierarchical structure with little grouping at the genus level, despite high bootstrap support for groupings in the concatenated sequence tree (supplementary fig. S4, Supplementary Material online).

The estimated gain and loss of both butyric acid production and sporulation functionality was mapped onto both the 16S and the concatenated sequence trees (fig. 4). Multiple acquisition points of each type of butyric acid production can be observed in both trees, supporting the case for LGT of this function into this group. However, if the 16S tree does map the true history of this group, or at least functions as a close proxy for vertical inheritance, the butyrate kinase gene (fig. 4*a*) may have been acquired through LGT by an ancestor of many of the family members and lost in three subsequent lineages, as opposed to five independent gains. This ancient LGT followed by loss in certain lineages is supported by the phylogenetic analysis of this gene, although directionality cannot be determined due to an unresolved species tree (supplementary fig. S1, Supplementary Material online). The observed pattern of sporulation capabilities (fig. 3) could be explained by four gene loss events, no matter the representative tree. This supports a model of vertical inheritance with subsequent gene loss in a habitat-specific manner. Additionally, within the 16S tree, most of the gut-restricted group (fig. 2) formed a near clade with one nongroup intruder (*Coprococcus comes* ATCC 27758) and one member absent (*Lachnospiraceae* bacterium 6_1_63FAA) (fig. 4).

## Discussion

Lachnospiraceae were found to be present primarily within the mammalian GI tract (fig. 1), as has been suggested previously (Gosalbes et al. 2011; Kittelmann et al. 2013), although low-abundance populations are present in a wider range of environments including nonhost-associated microbiomes. The capacity for butyric acid production, found in fewer than half of the Lachnospiraceae genomes, was not habitat restricted and appears to have been acquired through LGT. Both pathways for producing butyric acid (butyrate kinase and BCoAT) were present in Lachnospiraceae members, with no genome containing both (table 1). Although seven genomes contained butyrate kinase, they appear to have potentially acquired the corresponding gene laterally from other members of class Clostridia (supplementary fig. S1, Supplementary Material online), a group associated with frequent LGT events (Beiko et al. 2005; Sebaihia et al. 2006; Nelson et al. 2010), especially within GI tracts (Meehan and Beiko 2012). LGT has also contributed to the distribution of the BCoAT-mediated pathway, the main route for butyric acid production within the human GI tract (Louis et al. 2004; Louis and Flint 2009). Within both trees, the Lachnospiraceae-related sequences appear to form two clusters, suggesting that the LGT events that gave rise to these functions were likely prior to the speciation events for some of these organisms. Thus, although this function is not habitat-restricted presently, it may have conferred an ecological advantage to the ancestor of some present-day Lachnospiraceae. Determination of the donating partners is difficult in these cases as several species not designated as Lachnospiraceae in the NCBI taxonomy were found in close proximity to organisms such as *Roseburia* in the 16S phylogeny. An example is *E. rectale*, which Mannarelli et al. (1990) also placed in family Lachnospiraceae. Such discrepancies between published work and taxonomic databases make determination of directionality and evolutionary history difficult, as the species trees are not well resolved. Reconciling taxonomy and phylogeny is no trivial task given LGT and other challenges but would clarify the origin of butyrate production and other capabilities in the Lachnospiraceae.

Although butyric acid production was not found to segregate the Lachnospiraceae by habitat, several other functions were correlated with specific habitat-associated groups. Tetratricopeptide repeat motif-containing proteins were present in a subset of human GI tract-associated strains and absent from other members in the same environment (supplementary table S3a and S3b, Supplementary Material online). These motifs play a role in protein–protein interactions and have been associated previously with bacterial pathogens and virulence (Cerveny et al. 2013). As no Lachnospiraceae pathogens have been found before, further investigations into this group, which also lack butyric-acid production capabilities (supplementary table S4, Supplementary Material online), are needed to clarify their role or roles within the human gut.

The ability to produce endospores was found to be a habitat-specific segregating function within the Lachnospiraceae. Genome-wide investigation into the 22 Lachnospiraceae associated with the human GI tract revealed an almost full complement of sporulation proteins, whereas those residing in the human oral cavity or cow rumen were lacking such functions (fig. 3, supplementary table S3d, Supplementary Material online). *Cellulosilyticum lentocellum*, the only Lachnospiraceae with confirmed endospore formation capabilities (Attwood et al. 1996; Kelly et al. 2010), grouped with the GI tract-associated genomes. This strain was isolated from a sediment bank receiving domestic waste (Murray et al. 1986) and thus may actually be human associated with endospore formation as a habitat adaptation for passage through the human stomach as is observed in *C. difficile* (Wilson 1983) and cyst formation in several protist species (Bingham and Meyer 1979; Lujan et al. 1997). As analysis of these proteins suggested primarily vertical inheritance of the associated genes, it is likely that this capability was present in a common ancestor and subsequently lost in a habitat-specific fashion.

Our approach to understanding the Lachnospiraceae combined reference genomes of known provenance with marker gene and metagenome samples from a range of habitats. No phylogenomic approach we used produced a separation of lineages based on habitat, raising the question of how lineages can change their habitat preference through time. Discordance between the 16S tree and shared ortholog tree indicates that resolution of the “true” species tree may be very difficult for this group. Although a tree based on ribosome-related genes is often thought to be more accurate for species definitions than 16S alone, the Lachnospiraceae ribosomal protein trees were not in concordance with each other (supplementary fig. S5, Supplementary Material online), suggesting this approach will also give misleading results. We found little support for many genera within this family, and 16S trees placed several other organisms within this group (supplementary fig. S1, Supplementary Material online), suggesting taxonomic revisions may be required as has been done previously (Moon et al. 2008; Cai and Dong 2010). Despite the inconsistencies observed with regards to taxonomic classifications, some genes clearly separated lineages based on habitat. These genes shed light on how important habitat-specific transitions in the Lachnospiraceae have occurred and how within-habitat divisions, such as the ability to produce butyric acid, can influence the evolution of closely related organisms. As more Lachnospiraceae genomes become available covering important genera such as *Blautia* and likely mislabeled members such as *E. rectale*, similar analysis may reveal this pattern to extend to these genera and also potentially to other GI tract-associated microorganisms, revealing how such microbes adapt to the host environment.

## Supplementary Material

Supplementary figures S1–S5 and tables S1–S5 are available at *Genome Biology and Evolution* online (http://www.gbe.oxfordjournals.org/).

Supplementary Data
