# Supplementary material for: A Phylogenomic View of Ecological Specialization in the Lachnospiraceae, a Family of Digestive Tract-Associated Bacteria
Source: Genome Biol Evol. 2014 Mar 12;6(3):703–13. doi: 10.1093/gbe/evu050 (PMC3971600; doi:10.1093/gbe/evu050)
Supplement: Supplementary Data [file supp_6_3_703__index.html]

A Phylogenomic View of Ecological Specialization in the Lachnospiraceae, a Family of Digestive Tract-associated Bacteria — A Phylogenomic View of Ecological Specialization in the Lachnospiraceae, a Family of Digestive Tract-Associated Bacteria — Supplementary Data 

# A Phylogenomic View of Ecological Specialization in the Lachnospiraceae, a Family of Digestive Tract-Associated Bacteria

## Supplementary Data

files

**Files in this Data Supplement:**

- Supplementary Data - pdf file
- Supplementary Data - pdf file
- Supplementary Data - pdf file
- Supplementary Data - pdf file
- Supplementary Data - pdf file
- Supplementary Data - pdf file
- Supplementary Data - pdf file
- Supplementary Data - pdf file
- Supplementary Data - pdf file
- Supplementary Data - pdf file
