# Supplementary material for: A Phylogenomic View of Ecological Specialization in the Lachnospiraceae, a Family of Digestive Tract-Associated Bacteria
Source: Genome Biol Evol. 2014 Mar 12;6(3):703–13. doi: 10.1093/gbe/evu050 (PMC3971600; doi:10.1093/gbe/evu050)
Supplement: Supplementary Data [file supp_evu050_SuppFig1.pdf]

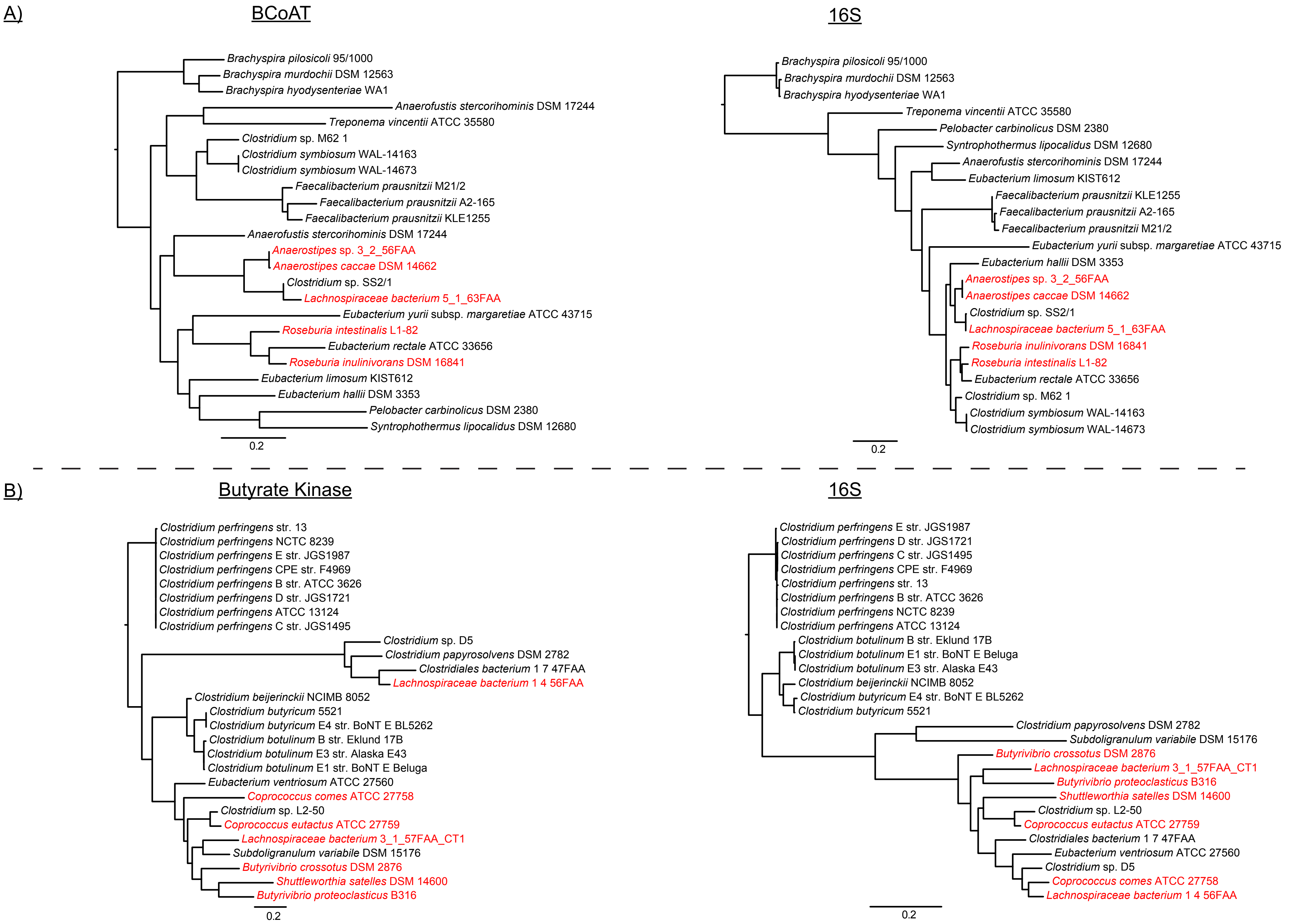

Supplementary figure S1 - Phylogenetic analysis of Lachnospiraceae-associated genes involved in the production of butyric acid and their associated 16S phylogenies.
The genes for butyryl-CoA:acetate CoA-transferase (A) and butyrate kinase (B) within Lachnospiraceae genomes were compared to 3,500 other prokaryotic genomes to find sources of potential LGT of these functions. Individual phylogenies were built using 16S sequences from genomes found to have the relevant butyrate-related gene and are displayed beside the BCoAT (A) and butyrate kinase (B) phylogenies. Lachnospiraceae members are highlighted in red.
