# Supplementary material for: A Phylogenomic View of Ecological Specialization in the Lachnospiraceae, a Family of Digestive Tract-Associated Bacteria
Source: Genome Biol Evol. 2014 Mar 12;6(3):703–13. doi: 10.1093/gbe/evu050 (PMC3971600; doi:10.1093/gbe/evu050)
Supplement: Supplementary Data [file supp_evu050_SuppTable5.pdf]

Supplementary Table S5- Functions of family-wise single-copy orthologs. Ninety-one single-copy orthologs were found to be shared amongst all Lachnospiraceae members. The functional description of each ortholog cluster is outlined below.

Ortholog Cluster	Description
1	16S rRNA methyltransferase GidB
2	30S ribosomal protein S5
3	30S ribosomal protein S10
4	30S ribosomal protein S11
5	30S ribosomal protein S12
6	30S ribosomal protein S13
7	30S ribosomal protein S15
8	30S ribosomal protein S2
9	30S ribosomal protein S7
10	30S ribosomal protein S8
11	50S ribosomal protein L1
12	50S ribosomal protein L10
13	50S ribosomal protein L13
14	50S ribosomal protein L19
15	50S ribosomal protein L23
16	50S ribosomal protein L4
17	50S ribosomal protein L9
18	50S ribosomal protein L11
19	50S ribosomal protein L14
20	50S ribosomal protein L15
21	50S ribosomal protein L16
22	50S ribosomal protein L18
23	50S ribosomal protein L2
24	50S ribosomal protein L20
25	50S ribosomal protein L22
26	50S ribosomal protein L27
27	50S ribosomal protein L29
28	50S ribosomal protein L30
29	50S ribosomal protein L5
30	50S ribosomal protein L6
31	50S ribosomal protein L7/L12
32	50S ribosomal protein L7A
33	ABC transporter ATP-binding protein
34	Alkaline shock protein 23
35	CDP-diacylglycerol--glycerol-3-phosphate 3-phosphatidyltransferase
36	DAK2 domain fusion protein YloV
37	DNA mismatch repair protein MutS
38	DNA-directed RNA polymerase
39	DNA-directed RNA polymerase
40	Elongation factor Tu
41	Exodeoxyribonuclease
42	Glycerol-3-phosphate dehydrogenase
43	GTP diphosphokinase
44	GTP-binding protein LepA
45	GTP-binding protein YchF
46	GTP-binding protein engA
47	GTP-binding protein engB
48	GTPase obg
49	Hemolysin A
50	Homoserine O-succinyltransferase
51	HPr kinase/phosphorylase
52	Hypothetical protein
53	Impact family protein
54	Iojap protein 155
55	KH domain protein
56	Leucyl-tRNA synthetase
57	Metalloprotease ybeY
58	O-sialoglycoprotein endopeptidase
59	P-loop ATPase
60	peptidyl-tRNA hydrolase
61	PhoH family protein
62	Phosphoglycerate kinase
63	Phosphopantetheine adenylyltransferase
64	Putative RNA-binding protein YqeI
65	Recombination protein recR
66	Ribonuclease III
67	Ribosome biogenesis GTP-binding protein YlqF
68	Ribosome recycling factor
69	RNA methylase family protein
70	S-adenosyl-methyltransferase MraW
71	S4 domain protein
72	Sigma-70, region 4
73	Signal recognition particle protein
74	Sporulation Regulator WhiA
75	SsrA-binding protein
76	Tetrapyrrole methylase
77	Transcription termination factor NusA
78	Transcription termination/antitermination factor NusG
79	Transcriptional regulator NrdR
80	Translation elongation factor Ts
81	Translation initiation factor IF-2
82	tRNA dimethylallyltransferase
83	tRNA uridine 5-carboxymethylaminomethyl modification enzyme GidA
84	tRNA-dihydrouridine synthase B
85	tRNA-guanine transglycosylase
86	Tyrosyl-tRNA synthetase
87	UDP-N-acetylenolpyruvoylglucosamine reductase
88	UMP kinase
89	UvrABC system protein B
90	Valyl-tRNA synthetase
91	YhgF protein
