# Supplementary material for: A Phylogenomic View of Ecological Specialization in the Lachnospiraceae, a Family of Digestive Tract-Associated Bacteria
Source: Genome Biol Evol. 2014 Mar 12;6(3):703–13. doi: 10.1093/gbe/evu050 (PMC3971600; doi:10.1093/gbe/evu050)
Supplement: Supplementary Data [file supp_evu050_SuppFig2.pdf]

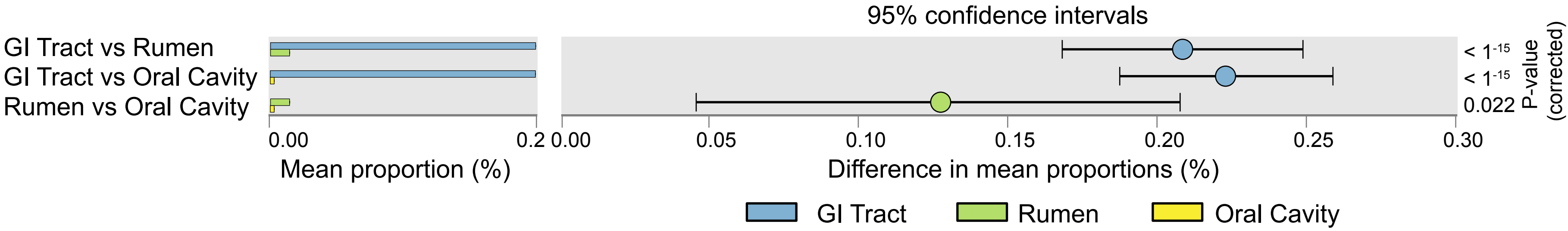

Supplementary figure S2- Sporulation-related sequences that differ in abundance between the human GI tract microbiome, and the cow rumen and human oral cavity. The abundance of reads assigned to Lachnospiraceae-associated sporulation genes within metagenomic samples from the human gut microbiome were compared to those within the cow rumen and the human oral cavity using STAMP. This revealed several genes that were more abundant in the human GI tract (blue) compared to the rumen (green) or oral cavity (yellow). The mean proportions of assigned reads within each dataset are shown in addition to the difference of these proportions between datasets. The p-value from the Bonferroni-corrected two-sided Welch's t-test is shown for each comparison.
