# Supplementary material for: A Phylogenomic View of Ecological Specialization in the Lachnospiraceae, a Family of Digestive Tract-Associated Bacteria
Source: Genome Biol Evol. 2014 Mar 12;6(3):703–13. doi: 10.1093/gbe/evu050 (PMC3971600; doi:10.1093/gbe/evu050)
Supplement: Supplementary Data [file supp_evu050_SuppFig3.pdf]

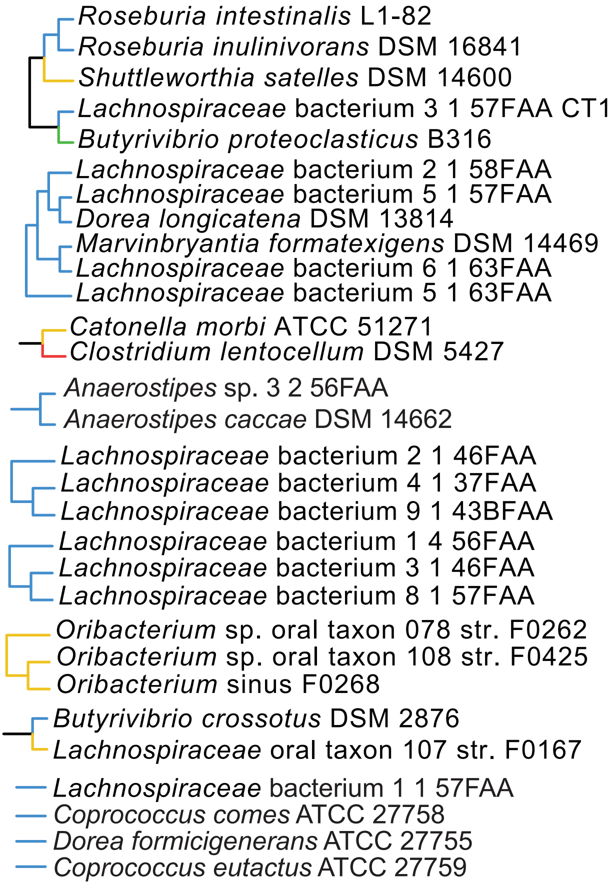

Supplementary figure S3 - Maximum agreement forest between the 16S and shared gene cluster phylogenetic trees.
SPR operations were used to assess the congruence of phylogenetic trees based upon the 16S gene and the shared gene clusters of all analyzed genomes. The maximum agreement forest displays components that are in present in both trees. Branches are colored based upon listed habitat (yellow = oral; red = sediment; green = rumen; blue = human GI tract).
