# Supplementary material for: A Phylogenomic View of Ecological Specialization in the Lachnospiraceae, a Family of Digestive Tract-Associated Bacteria
Source: Genome Biol Evol. 2014 Mar 12;6(3):703–13. doi: 10.1093/gbe/evu050 (PMC3971600; doi:10.1093/gbe/evu050)
Supplement: Supplementary Data [file supp_evu050_SuppFig4.pdf]

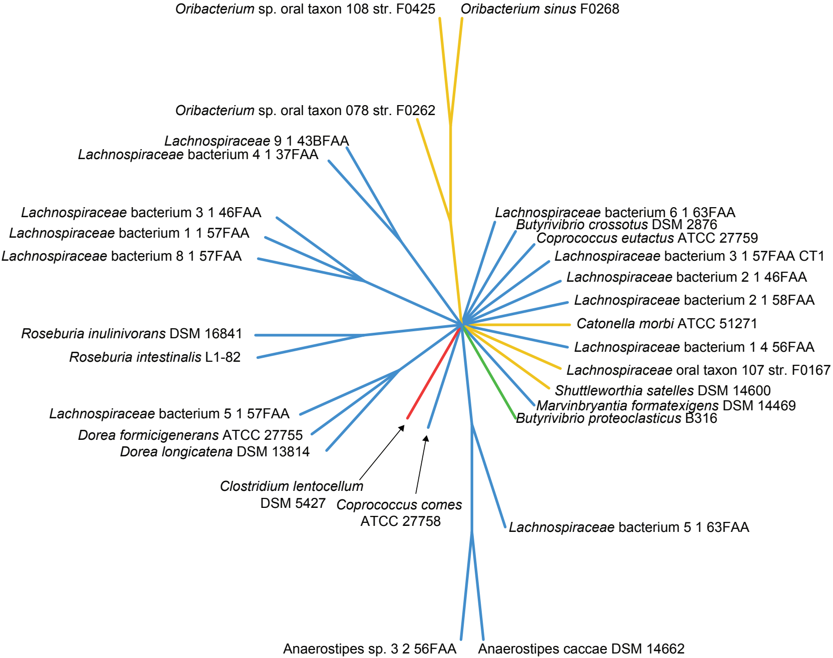

Supplementary figure S4- Phylogenetic network of shared gene clusters based upon individual gene tree topologies.
The gene trees of 91 family-wide shared gene clusters were input to SplitsTree4 to construct an unrooted phylogenetic network that best represented all the individual relationships. Most gene trees were found to disagree, resulting in a star-like topology. Branch coloring is based upon listed habitat (yellow = oral; red = sediment; green = rumen; blue = human GI tract).
