# Supplementary material for: A Phylogenomic View of Ecological Specialization in the Lachnospiraceae, a Family of Digestive Tract-Associated Bacteria
Source: Genome Biol Evol. 2014 Mar 12;6(3):703–13. doi: 10.1093/gbe/evu050 (PMC3971600; doi:10.1093/gbe/evu050)
Supplement: Supplementary Data [file supp_evu050_SuppFig5.pdf]

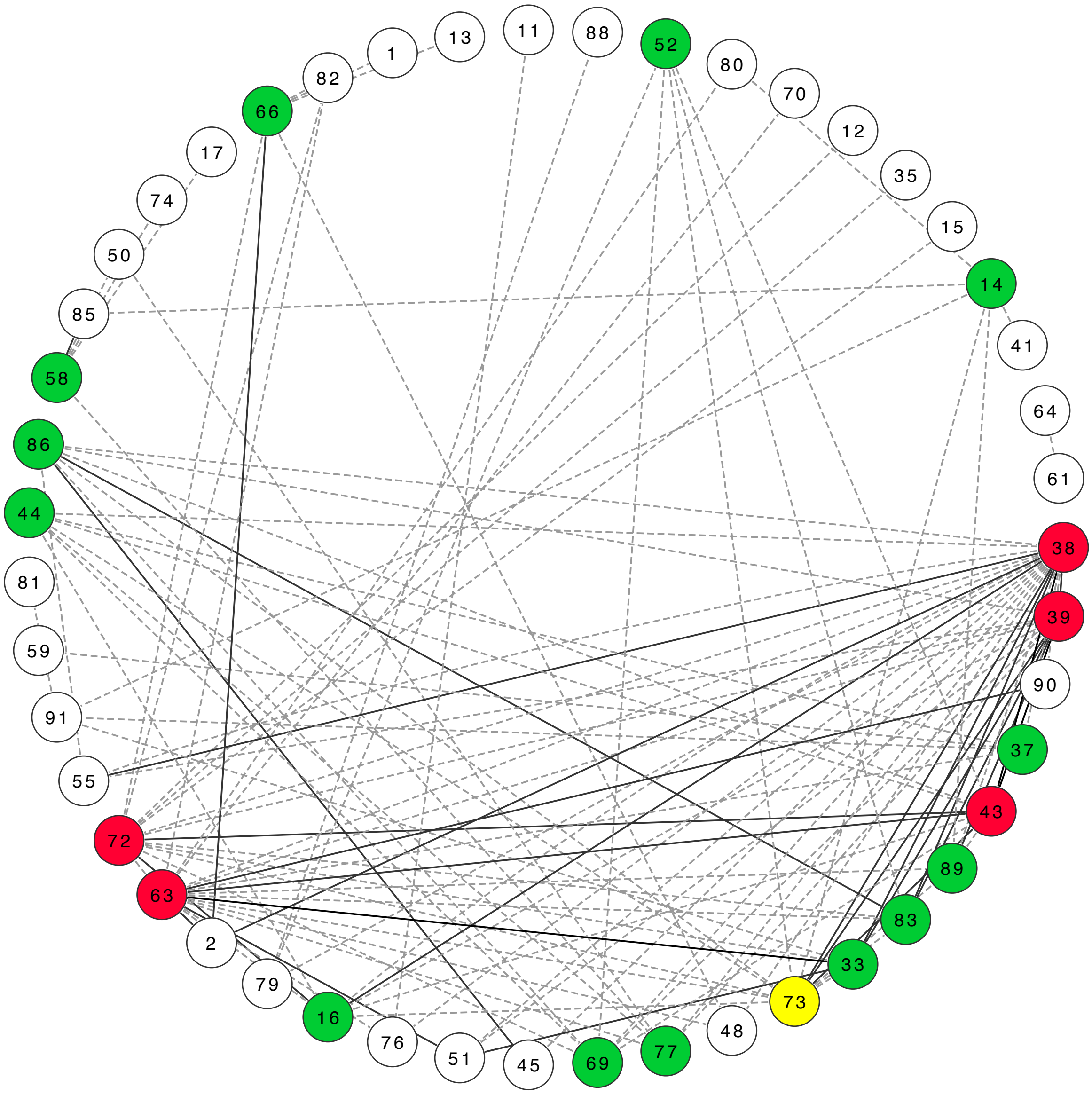

Supplementary figure S5- Similarity of single-copy ortholog gene trees.
The gene trees of the 91 single-copy orthologs were compared to each other by computing the rooted subtree prune-and-regraft (SPR) distance. Nodes connected by solid lines correspond to trees with an SPR distance of 0 or 1; nodes connected by a dashed line represent trees with a distance of 2. Nodes with over 10 connections are highlighted in red, those with between 5 and 10 connections are highlighted in green and below 5 in white. The 44 trees with SPR distance > 2 to all other trees are not shown. Ortholog cluster number correspond to those in Supplementary table S5.
