# Supplementary material for: A Phylogenomic View of Ecological Specialization in the Lachnospiraceae, a Family of Digestive Tract-Associated Bacteria
Source: Genome Biol Evol. 2014 Mar 12;6(3):703–13. doi: 10.1093/gbe/evu050 (PMC3971600; doi:10.1093/gbe/evu050)
Supplement: Supplementary Data [file supp_evu050_SuppTable1.pdf]

Supplementary table S1 - Metagenomic samples utilized for environmental distribution analysis.
Multiple habitat types were tested for the presence of Lachnospiraceae. Each habitat type is listed along with the MG-RAST ID of the project samples were retrieved from and the number of sample obtained from each project.

Habitat	Project Id	Sample Count
Air	74	11
Arsenic groundwater	70	21
Cow (G.I.)	504	1
Flea	75	252
Human (G.I.)	66	281
	133	18
	81	17
Human (non-Oral, non G.I.)	81	180
Human (oral)	81	18
Human infant (G.I.)	65	56
Human newborn (G.I.)	79	80
Hydrothermal vent	327	127
Mammal (other) (G.I.)	114	39
Mouse (G.I.)	245	181
	157	172
	83	38
Ocean	56	12
	57	12
	189	72
Plant	72	12
Sewage	122	16
Snake (G.I.)	77	109
Soil	67	26
	69	52
	71	48
	80	27
