# Supplementary material for: A Phylogenomic View of Ecological Specialization in the Lachnospiraceae, a Family of Digestive Tract-Associated Bacteria
Source: Genome Biol Evol. 2014 Mar 12;6(3):703–13. doi: 10.1093/gbe/evu050 (PMC3971600; doi:10.1093/gbe/evu050)
Supplement: Supplementary Data [file supp_evu050_SuppTable2.pdf]

Supplementary table S2 - Lachnospiraceae genomes.
The designation, abbreviation used in this manuscript, NCBI taxon identification number, associated habitat according to IMG and source of for each genome utilized in this study is listed.

Name	Abbreviated name	NCBI ID	Habitat	Reference
Anaerostipes caccae DSM 14662	*A. caccae*	411490	Human Digestive Tract	(S1)
Anaerostipes sp. 3_2_56FAA	*Anaerostipes 3_2_56*	665937	Human Digestive Tract	(S2)
Butyrivibrio crossotus DSM 2876	*B. crossotus*	511680	Human Digestive Tract	(S3)
Butyrivibrio proteoclasticus B316	*B. proteoclasticus*	515622	Cow Rumen	(S4)
Catonella morbi ATCC 51271	*C. morbi*	592026	Human Oral Cavity	(S5)
Cellulosilyticum lentocellum DSM 5427	*C. lentocellum*	642492	Estuarine mud bank	(S6)
Coprococcus comes ATCC 27758	*C. comes*	470146	Human Digestive Tract	(S7)
Coprococcus eutactus ATCC 27759	*C. eutactus*	411474	Human Digestive Tract	(S8)
Dorea formicigenerans ATCC 27755	*D. formicigenerans*	411461	Human Digestive Tract	(S9)
Dorea longicatena DSM 13814	*D. longicatena*	411462	Human Digestive Tract	(S10)
Lachnospiraceae bacterium 1_1_57FAA	*LAC* 1_1_57	658081	Human Digestive Tract	(S11)
Lachnospiraceae bacterium 1_4_56FAA	*LAC* 1_4_56	658655	Human Digestive Tract	(S12)
Lachnospiraceae bacterium 2_1_46FAA	*LAC* 2_1_46	742723	Human Digestive Tract	(S13)
Lachnospiraceae bacterium 2_1_58FAA	*LAC* 2_1_58	658082	Human Digestive Tract	(S14)
Lachnospiraceae bacterium 3_1_46FAA	*LAC* 3_1_46	665950	Human Digestive Tract	(S15)
Lachnospiraceae bacterium 3_1_57FAA_CT1	*LAC* 3_1_57FAA_CT1	658086	Human Digestive Tract	(S16)
Lachnospiraceae bacterium 4_1_37FAA	*LAC* 4_1_37	552395	Human Digestive Tract	(S17)
Lachnospiraceae bacterium 5_1_57FAA	*LAC* 5_1_57	658085	Human Digestive Tract	(S18)
Lachnospiraceae bacterium 5_1_63FAA	*LAC* 5_1_63	658089	Human Digestive Tract	(S19)
Lachnospiraceae bacterium 6_1_63FAA	*LAC* 6_1_63	658083	Human Digestive Tract	(S20)
Lachnospiraceae bacterium 8_1_57FAA	*LAC* 8_1_57	665951	Human Digestive Tract	(S21)
Lachnospiraceae bacterium 9_1_43BFAA	*LAC* 9_1_43B	658088	Human Digestive Tract	(S22)
Lachnospiraceae oral taxon 107 str. F0167	*LAC 107 F0167*	575593	Human Oral Cavity	(S23)
Marvinbryantia formatexigens DSM 14469	*M. formatexigens*	478749	Human Digestive Tract	(S24)
Oribacterium sinus F0268	*O. sinus*	585501	Human Oral Cavity	(S25)
Oribacterium sp. oral taxon 078 str. F0262	*Oribacterium 078*	608534	Human Oral Cavity	(S26)
Oribacterium sp. oral taxon 108 str. F0425	*Oribacterium 108*	904296	Human Oral Cavity	(S27)
Roseburia intestinalis L1-82	*R. intestinalis*	536231	Human Digestive Tract	(S28)
Roseburia inulinivorans DSM 16841	*R. inulinirans*	622312	Human Digestive Tract	(S29)
Shuttleworthia satelles DSM 14600	*S. satelles*	626523	Human Oral Cavity	(S30)

References for Supplementary table S2

(S1) Unpublished (see http://www.ncbi.nlm.nih.gov/genome/?term=txid411490)
(S2) Unpublished (see http://www.ncbi.nlm.nih.gov/genome/13727?project_id=61867)
(S3) Unpublished (see http://www.ncbi.nlm.nih.gov/genome/2083?project_id=55091)
(S4) Kelly WJ, et al. (2010) The glycobiome of the rumen bacterium Butyrivibrio proteoclasticus B316(T) highlights adaptation to a polysaccharide-rich environment. PloS one 5(8):e11942.
(S5) Unpublished (see http://www.ncbi.nlm.nih.gov/genome/1946?project_id=55757)
(S6) Miller DA, et al. (2011) Complete genome sequence of the cellulose-degrading bacterium Cellulosilyticum lentocellum. J Bacteriol 193(9):2357-2358
(S7) Unpublished (see http://www.ncbi.nlm.nih.gov/genome/967?project_id=54883)
(S8) Unpublished (see http://www.ncbi.nlm.nih.gov/genome/996?project_id=54541)
(S9) Unpublished (see http://www.ncbi.nlm.nih.gov/genome/2064?project_id=54513
(S10) Unpublished (see http://www.ncbi.nlm.nih.gov/genome/985?project_id=54515)
(S11) Unpublished (see http://www.ncbi.nlm.nih.gov/genome/2341?project_id=68209)
(S12) Unpublished (see http://www.ncbi.nlm.nih.gov/genome/2342?project_id=68205)
(S13) Unpublished (see http://www.ncbi.nlm.nih.gov/genome/2855?project_id=66429)
(S14) Unpublished (see http://www.ncbi.nlm.nih.gov/genome/2343?project_id=68203)
(S15) Unpublished (see http://www.ncbi.nlm.nih.gov/genome/2371?project_id=66427
(S16) Unpublished (see http://www.ncbi.nlm.nih.gov/genome/2344?project_id=68201)
(S17) Unpublished (see http://www.ncbi.nlm.nih.gov/genome/2526?project_id=63581)
(S18) Unpublished (see http://www.ncbi.nlm.nih.gov/genome/2346?project_id=68199)
(S19) Unpublished (see http://www.ncbi.nlm.nih.gov/genome/2347?project_id=61883)
(S20) Unpublished (see http://www.ncbi.nlm.nih.gov/genome/2350?project_id=66423)
(S21) Unpublished (see http://www.ncbi.nlm.nih.gov/genome/2372?project_id=61885)
(S22) Unpublished (see http://www.ncbi.nlm.nih.gov/genome/2352?project_id=66425)
(S23) Unpublished (see http://www.ncbi.nlm.nih.gov/genome/2556?project_id=66385)
(S24) Unpublished (see http://www.ncbi.nlm.nih.gov/genome/957?project_id=54943)
(S25) Unpublished (see http://www.ncbi.nlm.nih.gov/genome/13439?project_id=55891)
(S26) Unpublished (see http://www.ncbi.nlm.nih.gov/genome/13438?project_id=55773)
(S27) Unpublished (see http://www.ncbi.nlm.nih.gov/genome/13438?project_id=67819)
(S28) Unpublished (see http://www.ncbi.nlm.nih.gov/genome/2047?project_id=55267)
(S29) Unpublished (see http://www.ncbi.nlm.nih.gov/genome/2081?project_id=55375)
(S30) Unpublished (see http://www.ncbi.nlm.nih.gov/genome/1952?project_id=55775)
