# Supplementary material for: A Phylogenomic View of Ecological Specialization in the Lachnospiraceae, a Family of Digestive Tract-Associated Bacteria
Source: Genome Biol Evol. 2014 Mar 12;6(3):703–13. doi: 10.1093/gbe/evu050 (PMC3971600; doi:10.1093/gbe/evu050)
Supplement: Supplementary Data [file supp_evu050_SuppTable3.pdf]

Supplementary table S3 - Functions characterizing sub-groups of Lachnospiraceae.

Gene clusters present in over 90% of one group of Lachnospiraceae genomes and absent in over 90% of another were analyzed using Interproscan to determine their functions, as was the reverse. The general InterPro functional categories and GO

Supplementary table S3a. Gut-restricted Lachnospiraceae compared to all other Lachnospiraceae

Gut-restricted-associated genome count	Non-Gut-restricted genome count	InterPro	GO
11	0	Tetratricopeptide-like helical	Protein binding
		none	None
		TolB-like	Protein binding
		Tetratricopeptide-like helical	Protein binding
		None	None
11	1	Signal transduction histidine kinase	Signal Transduction
		None	none
		Peptidoglycan-binding Lysin subgroup	Cell wall macromolecule catabolic process
		Protein phosphatase 2C-like	Catalytic activity
		Periplasmic binding protein domain	None
		YbbR-like	None
		Type II secretion system F domain	None
		None	None
		None	None
		Colicin V production, CvpA	Toxin biosynthetic process
		Tetratricopeptide-like helical	Protein binding
		Aminoglycoside phosphotransferase	Transferring phosphorus-containing groups
		Tetratricopeptide-like helical	Protein binding
		None	None
		None	None
		None	None
		None	None
		Haemerythrin/HHE cation-binding motif	Metal ion binding
		none	None
		Spore cortex biosynthesis protein, YabQ-like	None
		none	None
11	2	Periplasmic binding protein domain	None
		Permease FtsX-like	None
		Sporulation stage III, protein AE	None
		Sporulation stage II protein D, amidase enhancer LytB	Sporulation resulting in formation of a cellular spore
		Integral membrane protein 1906	None
		Vitamin B12-dependent methionine synthase, activation domain	Methionine synthase activity
12	0	None	None
12	1	Bacterial periplasmic spermidine/putrescine-binding protein	Transporter activity
		Prokaryotic chromosome segregation/condensation protein MukB, N-terminal	Chromosome segregation
		Spore coat protein CotS	Transferase activity, transferring phosphorus-containing groups
		Nucleoside recognition Gate	Nucleoside binding
12	2	Peptidyl-prolyl cis-trans isomerase, PpiC-type	Isomerase activity
		Signal transduction histidine kinase	Signal transduction
		None	None
0	16	No clusters	No clusters
0	17	No clusters	No clusters
0	18	No clusters	No clusters
1	16	Binding-protein-dependent transport systems inner membrane component	Transport activity
1	17	No clusters	No clusters
1	18	No clusters	No clusters

Supplementary table S3b. Gut-restricted Lachnospiraceae compared to all other gut-associated Lachnospiraceae

Gut-restricted genome count	Other gut-associated genome count	InterPro	GO
11	0	Alcohol dehydrogenase, iron-type	Oxidoreductase activity
		Aminoglycoside phosphotransferase	Transferase activity, transferring phosphorus-containing groups
		Tetratricopeptide repeat	Protein binding
		Six-bladed beta-propeller, TolB-like	None
		Tetratricopeptide repeat	Protein binding
		None	None
		None	None
		None	None
11	1	Periplasmic binding protein domain	None
		Vacuolating cytotoxin	Pathogenesis
		Integral membrane protein 1906	None
		Signal transduction histidine kinase	Phosphorelay sensor kinase activity
		Peptidoglycan-binding lysin domain	Cell wall macromolecule catabolic process
		Protein phosphatase 2C (PP2C)-like	Catalytic activity
		Periplasmic binding protein domain	None
		YbbR-like	None
		Type II secretion system F domain	None
		Colicin V production, CvpA	Toxin biosynthetic process
		Tetratricopeptide repeat	Protein binding
		Tetratricopeptide repeat	Protein binding
		Haemerythrin-like, metal-binding domain	Metal ion binding
		Spore cortex biosynthesis protein, YabQ-like	None
		None	None
		None	None
		None	None
		None	None
		None	None
		None	None
		None	None
		None	None
		None	None
12	0	None	None
12	1	Bacterial periplasmic spermidine/putrescine-binding protein	Polyamine transport
		Spore coat protein CotS	None
		Nucleoside recognition Gate	Nucleoside binding
		None	None
0	9	Electron transfer flavoprotein, alpha subunit	Electron carrier activity
		Acyl-CoA oxidase/dehydrogenase	Acyl-CoA dehydrogenase activity
		Thiolase	Transferase activity
		Enoyl-CoA hydratase/isomerase, conserved site	Catalytic activity
0	10	No clusters	No clusters
1	9	Nitrogen regulatory protein PII	Regulation of nitrogen utilization
		Binding-protein-dependent transport systems inner membrane component	Transporter activity
		NUDIX hydrolase domain	Hydrolase activity
		Nitroreductase-like	Oxidoreductase activity
1	10	No clusters	No clusters

Supplementary table S3c. Functions associated with Lachnospiraceae within the human GI tract that can produce butyric acid compared to those lacking this capability

Gut-associated butyric acid producing genome count	Gut-associated non-butyric acid producing genome count	InterPro	GO
9	0	Electron transfer flavoprotein, alpha subunit	Flavin adenine dinucleotide binding
		Acyl-CoA dehydrogenase, conserved site	Oxidation-reduction process
		Thiolase	Transferase activity
		Crotonase superfamily	Metabolic process
9	1	Nitrogen regulatory protein PII	Enzyme regulator activity
10	0	No clusters	No clusters
10	1	No clusters	No clusters
0	11	No clusters	No clusters
0	12	No clusters	No clusters
1	11	Vacuolating cytotoxin	Pathogenesis
		Protein phosphatase 2C (PP2C)-like	Catalytic activity
		Haemerythrin-like, metal-binding domain	Metal ion binding
		None	None
1	12	Protein kinase-like domain	Transferase activity

Supplementary table S3d. All gut associated Lachnospiraceae compared to Lachnospiraceae from other habitats

Gut-associated genome count	Non-gut genome count	InterPro	GO
20	0	Sulfatase	Sulfuric ester hydrolase activity
		Replication protein, DnaD/DnaB domain	None
		Phosphoglycerate/bisphosphoglycerate mutase	Catalytic activity
		Calycin-like	None
		Sporulation protein YlmC/YmxH	None
20	1	Signal transduction histidine kinase	Signal transduction
		Signal transduction response regulator, receiver domain	Two-component signal transduction system (phosphorelay)
		Peptidase S8/S53, subtilisin/kexin/sedolisin	Serine-type endopeptidase activity
		Phospholipid/glycerol acyltransferase	Phospholipid biosynthetic process
		Spore coat assembly protein CotJB	None
		Folylpolyglutamate synthetase	Folic acid-containing compound biosynthetic process
		Phosphoribosyl-ATP pyrophosphohydrolase	Phosphoribosyl-AMP cyclohydrolase activity
		Uncharacterised protein family UPF0348	Catalytic activity
		PBP domain	None
		Nucleoside recognition Gate	Nucleoside binding
21	0	Heat shock protein DnaJ	Heat shock protein binding
		None	None
		Peptidase S11, D-alanyl-D-alanine carboxypeptidase A	Serine-type D-Ala-D-Ala carboxypeptidase activity
		Stage III sporulation protein AH-like	None
		FMN-binding	FMN binding
		Protein of unknown function DUF3792, transmembrane	None
		Stage III sporulation protein AC/AD family	None
		Sporulation protein YabP/YqfC	None
		Stage III sporulation protein AC	None
21	1	Multi antimicrobial extrusion protein	Drug transmembrane transporter activity
		PemK-like protein	DNA binding
		Transcription regulator HTH, GntR	Sequence-specific DNA binding transcription factor activity
		Catalase, manganese	Transition metal ion binding
		Small acid-soluble spore protein, alpha/beta-type	DNA topological change
		DNA helicase, UvrD/REP type	ATP-dependent DNA helicase activity
		Penicillin-binding protein, transpeptidase	Peptidoglycan-based cell wall biogenesis
		Peptidase S11, D-alanyl-D-alanine carboxypeptidase A	Serine-type D-Ala-D-Ala carboxypeptidase activity
		Sporulation stage III, protein AA	Nucleoside-triphosphatase activity
		Peptidase A25, germination protease	Spore germination
		Endodeoxyribonuclease IV	DNA repair
		ClpP/TepA	Serine-type endopeptidase activity
		Nucleoside recognition Gate	Nucleoside binding
		NIF system FeS cluster assembly, NifU, N-terminal	Iron-sulfur cluster assembly
		STAS domain	Regulation of transcription, DNA-dependent
		Sporulation protein YabP	None
22	0	Transposon-encoded protein TnpV	None
		Sporulation stage II, protein P	Protein binding
		RNA-binding S4 domain	RNA binding
		PhoU	None
		Stage V sporulation protein AA	None
22	1	RNA polymerase sigma-70 factor	Regulation of transcription, DNA-dependent
		RNA polymerase sigma-70 factor	Regulation of transcription, DNA-dependent
		Primosome PriB/single-strand DNA-binding	Single-stranded DNA binding
		Peptidase S55, sporulation stage IV, protein B	Protein binding
		Sporulation stage II protein D, amidase enhancer LytB	Metabolic process
		Stage V sporulation AD	Catalytic activity
		Cell wall hydrolase/autolysin, catalytic	Peptidoglycan catabolic process
		Sporulation stage V, protein T	None
		Protein of unknown function DUF177	None
		Sporulation stage V, protein AC	None
		Anti-sigma F factor	Protein serine/threonine kinase activity
		Sporulation stage V, protein AE	None
