# Supplementary material for: A Phylogenomic View of Ecological Specialization in the Lachnospiraceae, a Family of Digestive Tract-Associated Bacteria
Source: Genome Biol Evol. 2014 Mar 12;6(3):703–13. doi: 10.1093/gbe/evu050 (PMC3971600; doi:10.1093/gbe/evu050)
Supplement: Supplementary Data [file supp_evu050_SuppTable4.pdf]

Supplementary table S4 - Shared features between members of the human gut Lachnospiraceae listed as gut-restricted/non-gut-restricted and those either possessing or lacking the capability to produce butyric acid.

Lachnospiraceae residing in the human GI tract were classified in 2 ways: those classed as gut-restricted or not based upon shared gene clusters (Fig. 2) and those classed based upon their capability to produce butyric acid or not (Table 1). Overlap of species assigned as one or the other within each classification was identified, as were functions associated with each classification.

Gut-restricted	Butyric acid-producing	Species	Associated functions
No	Yes	*Anaerostipes 3_2_56*<br>*A. caccae*<br>*B. crossotus*<br>*C. comes*<br>*C. eutactus*<br>*LAC 3_1_57FAA_CT1*<br>*LAC 5_1_63*<br>*R. intestinalis*<br>*R. inulinivorans*	Acyl-CoA oxidase/dehydrogenase<br>Electron transfer flavoprotein, alpha subunit<br>Nitrogen regulatory protein PII<br>Thiolase
No	No	*M. formatexigens*	
Yes	Yes	*LAC 1_4_56*	
Yes	No	*D. formicigenerans*<br>*D. longicatena*<br>*LAC 1_1_57*<br>*LAC 2_1_46*<br>*LAC 2_1_58*<br>*LAC 3_1_46*<br>*LAC 4_1_37*<br>*LAC 5_1_57*<br>*LAC 6_1_63*<br>*LAC 8_1_57*<br>*LAC 9_1_43B*	Vacuolating cytotoxin<br>Protein phosphatase 2C (PP2C)-like<br>Haemerythrin-like, metal-binding domain
